# Photographs in Natural Colors

**Published:** 1881-07

**Authors:** 


### PHOTOGRAPHS IN NATURAL COLORS.

The announcement is again made that a process has been discovered for taking photographs possessing all the brilliancy and delicacy of the natural colors, and an exhibition of pictures thus naturally colored has just been held in London. According to the reports, the colors are produced by the action of light alone in the camera, and owe nothing whatever to the artist's brush. In the photographs exhibited, the coloring appeared to be quite true to nature, and delicate tones and shades were clear to the view. The flesh tint was exact to life, and full justice was done to gorgeous regimentals. The protruded tongue of a dog in one of the photographs possessed the exact color of nature. Some of the guests, says the *English Mechanic*, inspecting this collection, and not fully acquainted with the character of the latest invention, took it for granted that the work was done by skillful, artistic hands on ivory and other material, and could scarcely believe their eyes when informed that the color, as much as the form and outline, was produced by the light of day. Careful investigation, however, would then show that human handicraft was not in it; for there were touches and effects which Nature's pencil of light could alone accomplish. The contention is that photographs colored by artists, however clever, must be more or less "monotonous, hard, untrue to Nature, and to the originals."

The process was discovered, it is said, by a French scientist, but has since undergone improvement by the proprietor of the process in England. If the new system proves an unqualified success, the reward will not have been reaped without much labor in the past, for numerous attempts have been made to induce the sun-pencil to fix colors in the picture it draws in the camera ; but chemical and mechanical difficulties have stood in the way. In the new process colors are said not only to be faithfully produced, but protected from the action of light by being passed through a boiling solution, of which gelatine forms the principal ingredient, and that some of the photographs so treated have been exposed for months to the sun without being in anywise affected by the ordeal. Unfortunately the process is yet unknown, as it is likely to be for some time to come.—*Manufacturer and Builder.*

